# Prevention of seroma following inguinal lymph node dissection with prophylactic, incisional, negative-pressure wound therapy (SEROMA trial): study protocol for a randomized controlled trial

**DOI:** 10.1186/s13063-018-2757-6

**Published:** 2018-08-15

**Authors:** Mads Gustaf Jørgensen, Navid Mohammadpour Toyserkani, Nana Hyldig, Annette Hougaard Chakera, Lisbet Rosenkrantz Hölmich, Jørn Bo Thomsen, Jens Ahm Sørensen

**Affiliations:** 1 Department of Plastic Surgery, Odense University Hospital, Sdr. Boulevard 29, 5000 Odense, Denmark; 2 Hans Christian Andersen’s Children Hospital, Odense University Hospital, Odense, Denmark; 3 Department of Plastic Surgery, Herlev Gentofte Hospital, Herlev, Denmark; 4 Department of Plastic Surgery, Roskilde Hospital, Roskilde, Denmark

**Keywords:** Prevention, Negative-pressure wound therapy, Seroma, Surgical-site infection, Lymphedema, Lymph node dissection, Melanoma

## Abstract

**Background:**

Radical inguinal lymphadenectomy (ILND) for metastatic melanoma is associated with a high complication rate. Seroma is often the first postoperative complication, followed by prolonged wound healing sometimes requiring reoperation, infection, multiple outpatient visits and re-hospitalization. Prevention of seroma may, therefore, lead to a reduction in many of the other complications.

**Methods/design:**

The primary aim of this randomized study is to investigate whether fewer patients require treatment for seroma by immediate prophylactic application of incisional, Negative-pressure Wound Therapy (iNPWT) following ILND, compared to standard postoperative treatment. The secondary outcomes include surgical-site infection, dehiscence, hematoma, length of hospitalization, quality of life, safety, long-term assessment of lymphedema and non-inferiority oncological outcome. Data will be registered prospectively at check-ups after 7 and 14 days, 1 and 3 months and 2 years after inguinal lymphadenectomy using case report forms and questionnaires and stored in a secure online database.

**Discussion:**

To our knowledge, this trial is the first randomized study evaluating negative-pressure wound therapy as a prophylactic intervention for complications following melanoma-related ILND. The results from this trial will hopefully determine the efficacy and safety of prophylactic iNPWT treatment in prevention of the clinical relevant short- and long-term postoperative complications following ILND and may provide an evidence base for the an improved postoperative regimen.

**Trial registration:**

ClinicalTrials.gov, ID: NCT03433937. Prospectively registered on 15 February 2018.

**Electronic supplementary material:**

The online version of this article (10.1186/s13063-018-2757-6) contains supplementary material, which is available to authorized users.

## Background

The incidence of malignant melanoma is rising. If malignant melanoma spreads, it is most commonly to the regional lymph nodes [1]. Inguinal or ilioinguinal lymph node dissection (ILND) is an operative intervention, where the lymphatic tissue and surrounding fat is removed from the groin en bloc to try to prevent further dissemination of the melanoma [2]. In case of verified or suspected metastasis to the pelvis, lymph nodes inferior to the internal iliac artery are also removed. However, the procedure is associated with a significant morbidity rate [3]. Studies have shown that up to 80% of patients undergoing ILND experience major postoperative complications such as seroma, surgical site infection (SSI), wound breakdown, hematoma and lymphedema [4]. Seroma is a collection of serous fluid in the wound cavity, formed by inflammatory exudates in response to the acute phase of wound healing following surgical trauma and/or by leaking lymph vessels [5]. Postoperative seroma is seen in about 50% of patient undergoing ILND [4], and is, therefore, one of the most frequently encountered complications. The serous fluid accumulation distends the wound cavity leading to increased risk of SSI, prolonged wound healing and possibly leading to higher risk of lymphedema [3]. Patients suffering from seroma and related wound complications often require prolonged hospitalization and multiple outpatient visits with associated high costs to the health system [6, 7]. Due to the high complication rate following ILND [8], there is a need for new evidence-based strategies to reduce the risk of complications.

Incisional Negative-pressure Wound Therapy (iNPWT) is a mechanical treatment modality, which has been shown to facilitate wound healing through vacuum-assisted closure [9]. The vacuum package consists of a single-use, battery-powered device and a dressing, which can be applied to the surgical field after the incision has been closed. Biomechanical studies have shown that iNPWT treatment leads to removal of interstitial fluid and increased blood and lymph flow which may in turn decreases the risk of seroma and lymphedema [10–12]. The risk of postoperative seroma and SSI following other surgical procedures has been shown to be halved by the prophylactic use of iNPWT [13]. However, it remains to be investigated as to whether the same technique can be used to reduce complications after ILND.

The aim of this study is to investigate the value of iNPWT in reducing complications following ILND, when applied to the surgical field immediately after macroscopic complete removal of all malignancy [14, 15].

## Methods/design

### Study objective

The aim of this study is to investigate whether patients undergoing ILND due to lymph node metastasis from melanoma will benefit from prophylactic iNPWT (Smith&Nephew). The primary aim is to evaluate whether patients treated with iNPWT will experience less seroma formation than patients treated with standard dressing, measured by the need of seroma aspirations by skin puncture. The secondary aims are to examine whether the use of iNPWT results in a reduction of other major complications, length of hospitalization, and improved patient quality of life. The safety of iNPWT in the oncological setting will be evaluated by comparing the distribution of patients with regional recurrence between groups.

### Sample size

It is estimated that at least 55% of patients undergoing ILND require intervention due to postoperative seroma [3], and we hypothesize that NPWT will reduce the number of patients needing seroma aspirations by 50%. A two-sided sample size calculation with 0.8 power and significance level of 0.05 reveals that we will have to include 100 patients. With an estimated dropout rate of 10%, we will need a sample size of 110 patients with 55 patients in the treatment and control groups, respectively. Sample size was calculated using STATA, version 14.0 (StataCorp, College Station, TX, USA) using the two-sample proportion test.

### Trial design

**Fig. 1:**
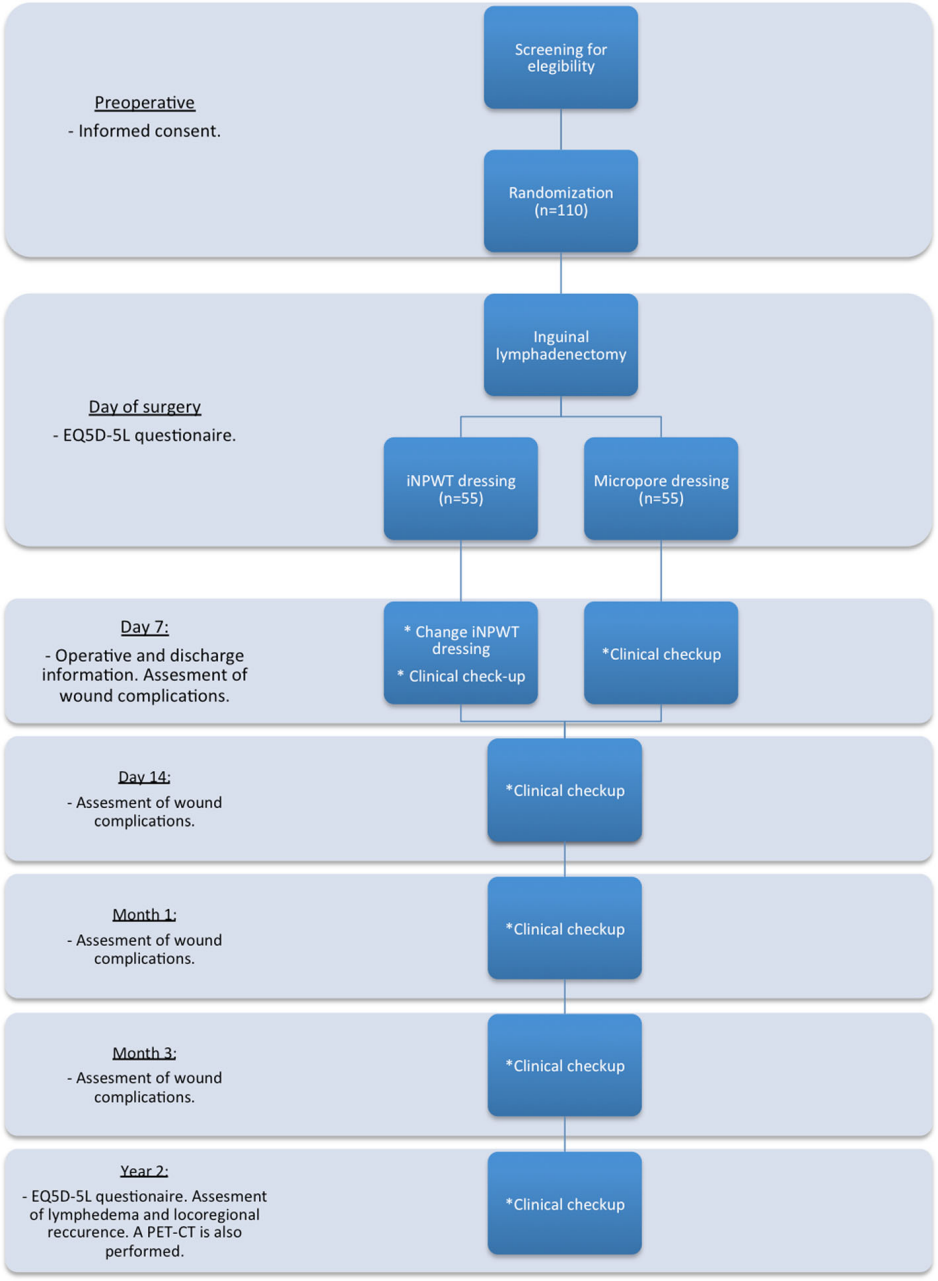
Screening, treatment and follow-up algorithm. Overview of the trial process

The study is a prospective, randomized, open-labeled, multicenter trial with two parallel groups (Fig. 1). Patients who have given informed consent to participate in the study will be randomized via a computer randomization program to either prophylactic iNPWT or a standard postoperative dressing (Micropore™ tape, 3 M, Copenhagen, Denmark) in a 1:1 allocation. If a patient requires bilateral ILND, both sides will be randomized individually. Included patients will initially be followed for 3 months for monitoring of the early postoperative complications. Any complications, both short-term complications, lymphedema and cancer recurrence will be dealt with at time of occurrence according to institutional protocol and national guidelines [16]. At the 2-year follow-up, the patient’s lower limbs will be clinically assessed for lymphedema using the International Society of Lymphology consensus [17]. Local cancer recurrence in the operated groin will be calculated by 2 years of follow-up. Trial participants will not receive any compensation or remuneration for their participation in the trial. This study is reported in accordance with the Standard Protocol Items: Recommendations for Interventional Trials (SPIRIT) Checklist for clinical trial protocols (Additional file 1) [18].

### Research ethics approval and data management

The trial has been approved by The Regional Committees on Health Research Ethics (S-20170085) and registered with the Danish Data Protection Agency (2008–58-0018).

All protocol-required information on each participant is recorded in an electronic case report form, using the secure online system REDCap [19] (https://www.project-redcap.org/). The entered data will be stored on a secure server in the Region of Southern Denmark via Odense Patient data Explorative Network (https://open.rsyd.dk).

### Study setting

The study will be conducted at the Department of Plastic and Reconstructive Surgery, Odense University Hospital, Denmark, the Department of Plastic and Reconstructive Surgery, Herlev Gentofte Hospital, Denmark and the Department of Plastic and Reconstructive Surgery, Roskilde Hospital, Denmark. Additional study centers may be included depending on logistics and feasibility.

### Eligibility criteria

Eligible patients will be recruited at undisturbed consultations and patients will not be contacted about the study prior to this appointment. Patients are recruited either during their follow-up course after skin melanoma treatment where a metastasis to the groin area can be identified clinically, by ultrasonography or PET-CT scan and a subsequent biopsy will confirm the diagnosis. The patient may also present with metastatic disease in the groin at initial consultation. ILND will only be offered, if there is no evidence of disease elsewhere and all such patients will be discussed at a multi-disciplinary team meeting according to national guidelines. Completion lymph node dissection for micrometastatic disease diagnosed with sentinel node biopsy is by 2018 no longer performed routinely in Denmark [16].

At the study centers, melanoma patients aged 18 years or older referred for ILND will be screened for trial eligibility. Before enrollment, patients will receive oral and written information about the study and a folder explaining their legal rights. Patients will be excluded from the study if they are suspected of having residual tumor in the groin following ILND, have received previous groin irradiation or are suffering from dementia or any psychiatric disorder making them incapable of informed consent or adherence to follow-up. Furthermore, patients unable to communicate in Danish or English will be excluded from the study.

### Interventions

The ILND is performed by surgical removal of all lymph nodes and adipose tissue in the triangular region delineated by the sartorius muscle, adductor longus muscle and the inguinal ligament. At the end of the procedure, one or two suction drains are placed from the surgical cavity though the subcutis and skin distally or lateral from the inguinal wound, and anchored using a 3.0 nylon suture. All patients receive prophylactic, intravenous, perioperative antibiotic according to allergy and institutional protocol. The wound is then closed using an absorbable 3.0 vicryl suture and a 4.0 nylon or monofilament absorbable suture and covered with an iNPWT dressing or Micropore™ tape, depending on treatment arm. The suction drain(s) is placed in a manner, which allows the iNPWT to cover the surgical field in all cases. The iNPWT dressing will be used for a total of 14 days, and the dressing will be changed on the seventh postoperative day. After removal of the iNPWT dressing, Micropore™ dressing is then optionally used to cover the scar for up to 3 months postoperatively for all patients. Patients in the control arm receive standard postoperative wound dressing consisting of optional Micropore™ dressing for up to 3 months. The drain(s) are to be removed at the seventh postoperative day or when there is a daily output of less than 20 mL per drain, whichever occurs first.

### Follow-up and data collection methods

Clinical follow-up data and short-term complications will be analyzed from the day of surgery up until 3 months after surgery. Patients will be seen at clinical visits at day 7 and 14, as well as 1 and 3 months postoperatively. Data will be collected prospectively at every check-up and registered in a case report form, whether or not an outcome has occurred since the last check-up. For registration of events outside of scheduled follow-up visits, patients receive a handout case report form and are instructed to bring the form, when in contact with any health practitioner. Daily drain output will be registered on a separate case report form until drain removal by the department staff. Two years after allocation, patients will be invited for an additional check-up and examined for lymphedema occurrence and regional recurrence.

In Denmark, stage III melanoma patients are enrolled in a national follow-up program consisting of clinical examination every third month for the first 2 years, and every sixth months for 3 years. After 5 years of hospital follow-up, a yearly visit with the general practitioner is advised until 10 years after initial cancer treatment. In addition, the patients undergo routine PET-CT scans after 6, 12, 24 and 36 months.

**Fig. 2:**
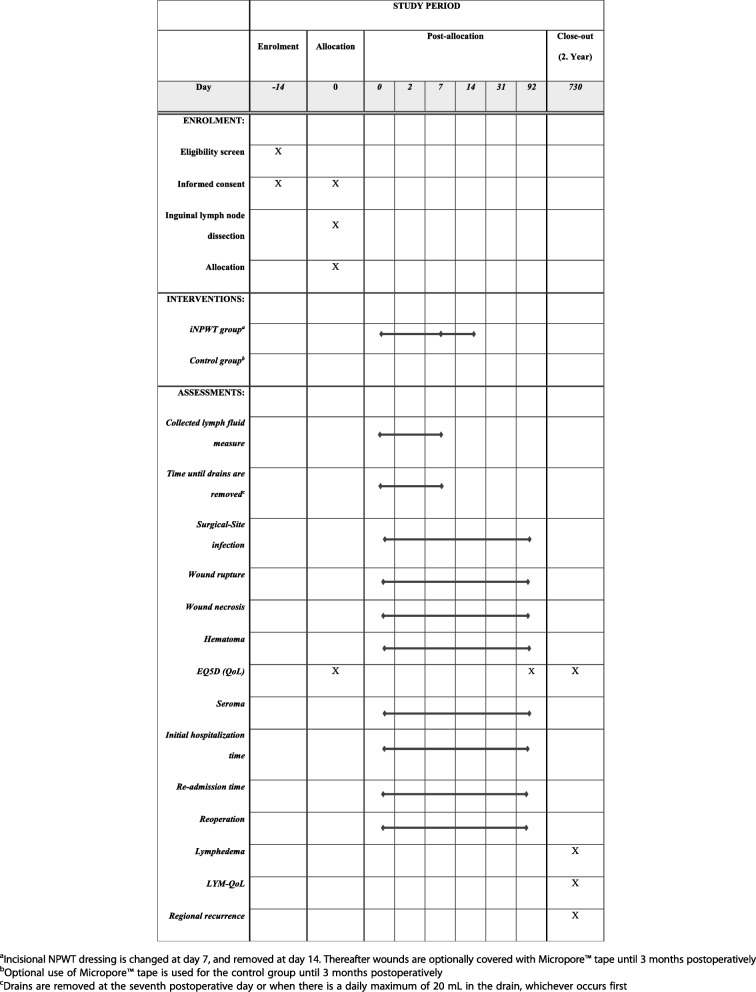
Participant timelines

Patients lost to follow-up will be contacted for outcome registration and electronic medical records will be reviewed to identify missing data (Fig. 2).

### Outcomes

The primary outcome is the number of patients requiring seroma aspiration. For this study, seroma is defined as all clinical recognized and punctured seromas, which have an aspirated volume of at least 30 mL.

Secondary outcomes include the:

- Cumulative volume of aspirated seromas measured in milliliters after aspiration. Timeframe: registered until 3 months after ILND
- Cumulative number of seroma aspirations fulfilling above criteria. Timeframe: registered until 3 months after ILND
- Surgical site infection, defined as groin infection requiring antibiotic treatment. Timeframe: registered until 3 months after ILND
- Days until the last suction drain(s) is removed. Timeframe: maximum 7 days
- Cumulative volume of collected lymph fluid accumulated in the suction drains measured in milliliters. Timeframe: registered until last drain removal
- Health-related quality of life, assessed using the validated EQ-5D-5 L [20] questionnaire. Timeframe: assessed on the day of surgery, after 3 month and at the final check-up 2 years after ILND
- Wound dehiscence, defined as a wound edge dehiscence that requires secondary suturing or NPWT treatment. Timeframe: registered until 3 months after ILND
- Necrosis of the wound, defined as the presence of dead tissue, which require debridement on the attending physician’s discretion. Timeframe: registered until 3 months after ILND
- Hematoma, defined as an inguinal surgical cavity filled by blood or clots, which require evacuation. Timeframe: registered until 3 months after ILND
- Length of hospitalization, defined as the number of days from ILND until discharge. Timeframe: registered until 3 months after ILND
- Re-admission times, defined as the cumulative number of days that patients are re-admitted to the ward or have inpatient visits for reasons relating to the surgical site. Timeframe: registered until 3 months after ILND
- Reoperation, defined as any adverse complication (e.g., deep infection, hematoma, wound dehiscence, necrosis, continuous lymph leakage) from the inguinal wound, resulting in the patient undergoing a re-operation with opening of the wound/scar under general anesthesia or wound revision with skin graft or NPWT. Timeframe: registered until 3 months after ILND
- Lymphedema, defined as a clinical stage 1 or more on the International Society of Lymphology clinical grading scale [17]. Timeframe: registered at the 2-year check-up after ILND
- Lymphedema-related quality of life, assed using the LYM-QOL questionnaire [21]. Timeframe: registered at the 2-year check-up after ILND
- Regional recurrence of melanoma, defined as any melanoma recurrence in the groin verified by pathology. Timeframe: evaluated by the 2-year check-up after ILND. However, any recurrence will be treated surgically or oncologically according to national guidelines at the time of occurrence

Contact information, demographic data and data related to excised lymph nodes will be collected prospectively for all patients, including age, sex, smoking, alcohol consumption, Body Mass Index (BMI), diabetes, total number of lymph nodes removed, number of metastatic lymph nodes, size and location of metastases in the lymph nodes, number of lymph nodes with perinodal tumor growth and if the procedure also involved dissection of iliac lymph nodes.

## Statistical analysis

All analysis will be conducted on the intention-to-treat principle using STATA (StataCorp, College Station, TX, USA). Baseline variables are used to describe the characteristics of the trial participants. Continuous variables are summarized as mean and standard deviation or as median and interquartile range (25th to 75th percentiles) if the distribution is asymmetrical. Categorical variables are summarized as numbers and percentages. The categorical variables will be compared between groups with chi-squared test or Fisher’s exact test depending on the number of events. The continuous variables will be compared between groups using an unpaired *t* test or Mann-Whitney depending on data representation. A two-sided *P* value of less than 0.05 will be considered significant and reported with a 95% confidence interval when applicable

The primary outcome is the proportion of patients with at least one seroma aspiration and will be compared between groups using chi-squared test or Fisher’s exact test, depending on the number of events.

The secondary outcomes consist of categorical and continuous variables. SSI, wound dehiscence, wound necrosis, hematoma, reoperation, lymphedema and regional recurrence will be sampled as categorical variables and compared between groups. The cumulative volume and number of seroma aspirations, volume of collected lymph fluid, days until drain removal, quality of life scores, length of hospitalization and re-admission times will be registered as continuous variables and compared between groups.

Subgroup analyses of patients with iliac LND will be conducted as removal of these more proximal and deeper lymph nodes require extensive surgery beyond the inguinal boundary. Patients requiring this additional surgery may be at an increased risk of additional morbidity such as lymphedema, and recurrence.

## Discussion

The purpose of this trial is to evaluate the efficacy and oncological safety of iNPWT for the prevention of the clinically relevant morbidities after ILND. In addition, this study is an advancement of trial methodology for assessment of postoperative outcomes after lymph node excisions. The study is expected to generate valuable new information in the possible prevention of postoperative complications and improve recovery following cancer treatment.

Lymph node dissection is associated with considerable morbidity and selection of appropriate patients is of importance. For melanoma patients with a low lymph-node-tumor burden (micrometastases in sentinel nodes), the MSLT-II trial showed no additional 3-year survival for complete dissection when compared to observation and delayed lymph node dissection in case the patient later revealed regional metastases [2]. While a long-term survival comparison is still pending, complete dissection provides better regional disease control and could be advocated for patients with a greater tumor burden, and the general opinion is that patients with larger metastases should still undergo lymph node dissection if no evidence of distant metastases. Patients undergoing dissection of the groin lymph nodes are particularly prone to postoperative morbidity and previous studies have suggested conservative and minimally invasive ILND protocols [22, 23] however, these have yet to achieve broad acceptance due to the risk of compromising oncological safety. In this study, the ILND procedure is performed according to the national guidelines in Denmark, and, therefore, does not vary between study groups. Because iNPWT may increase tissue vascularization, the device is only applied when all malignancy has been excised. Thus, it is presumed that there will be no difference in regional recurrence between intervention and control groups. The safety and tolerability of iNPWT for other indications has been well established for many years. Reported discomforts related to its use has been associated to bandage removal, which is similar to the discomfort following regular postoperative dressing removal [13, 24]. This minor discomfort is, however, insignificant compared to the potential benefit of iNPWT application.

The outcomes investigated in this study were chosen due to their relevance to clinical practice and postoperative complications were defined as the need for treatment. The primary outcome, seroma, is often the first sign of a problematic wound-healing trail and prevention may, therefore, in turn, prevent some of the frequent subsequent complications. During the planning of the study, blinding and the use of a sham iNPWT device was considered, but not found feasible due to the nature of the intervention, which exerts a vacuum on the wound. The study will be conducted in accordance with the published protocol and positive, negative and inconclusive results on the short-term complications will be published, when all patients have completed their 3-month follow-up visits. A separate publication on the long-term lymphedema evaluation and regional recurrence will be published, when all patients have completed their 2-year follow-up. All publications derived from this study will adhere to the Vancouver author convention.

Prevention of diseases can be more efficient than treatment if the preventive intervention is more cost-effectiveness than the treatment [25, 26]. In this study, a relatively inexpensive device may prevent several outpatient and inpatient appointments and treatment-related costs. The results from this trial will, therefore, elucidate the efficacy and safety of prophylactic iNPWT treatment in prevention of the major short- and long-term postoperative complications following ILND. Regardless of the outcome, this study will benefit the patients by providing a solid foundation for future research in the field of prophylactic wound care treatment after lymph node dissection for cancer in general, possibly also in other regions such as the axilla and the neck.

## Trial status

At the time of manuscript submission, the trial is not yet actively enrolling participants.

## Additional file

Additional file 1: Standard Protocol Items: Recommendations for Interventional Trials (SPIRIT) 2013 Checklist: recommended items to address in a clinical trial protocol and related documents*. (DOC 239 kb)

## Abbreviations

ILND: Radical inguinal lymphadenectomy
iNPWT: Incisional, Negative-pressure Wound Therapy
SSI: Surgical-site infection
