# Correction to: Prevention of seroma following inguinal lymph node dissection with prophylactic, incisional, negative-pressure wound therapy (SEROMA trial): study protocol for a randomized controlled trial

**DOI:** 10.1186/s13063-018-2882-2

**Published:** 2018-10-19

**Authors:** Mads Gustaf Jørgensen, Navid Mohamadpour Toyserkani, Nana Hyldig, Annette Hougaard Chakera, Lisbet Rosenkrantz Hölmich, Jørn Bo Thomsen, Jens Ahm Sørensen

**Affiliations:** 1 Department of Plastic Surgery, Odense University Hospital, Sdr. Boulevard 29, 5000 Odense, Denmark; 2 Hans Christian Andersen’s Children Hospital, Odense University Hospital, Odense, Denmark; 3 Department of Plastic Surgery, Herlev Gentofte Hospital, Herlev, Denmark; 4 Department of Plastic Surgery, Roskilde Hospital, Roskilde, Denmark

## Correction

Following publication of the original article [1], the authors reported that one of the authors’ names is spelled incorrectly. In this Correction the incorrect and correct author name are shown. The original publication of this article has been corrected.

Originally the author name has been published as:

- Navid Mohammadpour Toyserkani

The correct author name is:

- Navid Mohamadpour Toyserkani
